# Polycarbonate mPOF-Based Mach–Zehnder Interferometer for Temperature and Strain Measurement

**DOI:** 10.3390/s20226643

**Published:** 2020-11-20

**Authors:** Xiaoyu Yue, Haijin Chen, Hang Qu, Rui Min, Getinet Woyessa, Ole Bang, Xuehao Hu

**Affiliations:** 1 Research Center for Advanced Optics and Photoelectronics, Department of Physics, College of Science, Shantou University, Shantou 515063, China; 19xyyue@stu.edu.cn (X.Y.); 18hjchen1@stu.edu.cn (H.C.); haqux@stu.edu.cn (H.Q.); 2 Key Laboratory of Intelligent Manufacturing Technology of MOE, Shantou University, Shantou 515063, China; 3 Center for Cognition and Neuroergonomics, State Key Laboratory of Cognitive Neuroscience and Learning, Beijing Normal University at Zhuhai, Zhuhai 519087, China; ruimin@bnu.edu.cn; 4 DTU Fotonik, Department of Photonics Engineering, Technical University of Denmark, 2800 Kgs. Lyngby, Denmark; gewoy@fotonik.dtu.dk (G.W.); oban@fotonik.dtu.dk (O.B.)

**Keywords:** polymer optical fibers, optical fiber devices, butt-coupling, Mach-Zehnder interferometer, temperature, strain

## Abstract

In this paper, an endlessly single mode microstructured polymer optical fiber (mPOF) in a Mach–Zehnder (M–Z) interferometer configuration is demonstrated for temperature and strain measurement. Because there is no commercial splicer applied for POF-silica optical fiber (SOF) connectorization, prior to the M–Z interferometric sensing, we introduce an imaging projecting method to align a polycarbonate mPOF to a SOF and then the splice is cured permanently using ultraviolet (UV) glue. A He-Ne laser beam at 632.8 nm coupled in a SOF is divided by a 1 × 2 fiber coupler to propagate in two fiber arms. A piece of mPOF is inserted in one arm for sensing implementation and the interference fringes are monitored by a camera. For non-annealed fiber, the temperature sensitivity is found to be 25.5 fringes/°C for increasing temperature and 20.6 fringes/°C for decreasing temperature. The converted sensitivity per unit length is 135.6 fringes/°C/m for increasing temperature, which is twice as much as the silica fiber, or 852.2 rad/°C/m (optical phase change versus fiber temperature), which is more than four times as much as that for the PMMA fiber. To solve the sensitivity disagreement, the fiber was annealed at 125 °C for 36 h. Just after the thermal treatment, the temperature measurement was conducted with sensitivities of 16.8 fringes/°C and 21.3 fringes/°C for increasing and decreasing process, respectively. One month after annealing, the linear response was improved showing a temperature sensitivity of ~20.7 fringes/°C in forward and reverse temperature measurement. For the strain measurement based on non-annealed fiber, the sensitivity was found to be ~1463 fringes/%ε showing repeatable linear response for forward and reverse strain. The fiber axial force sensitivity was calculated to be ~2886 fringes/N, showing a force measurement resolution of ~3.47 × 10^−4^ N. The sensing methodology adopted in this work shows several advantages, such as very low cost, high sensitivity, a straightforward sensing mechanism, and ease of fabrication.

## 1. Introduction

Polymer optical fibers (POFs) offer attractive advantages compared to their silica counterparts. POFs normally feature smaller Young’s modulus, larger thermo-optic coefficient and excellent biocompatibility, which makes them good candidates for sensing of temperature [1], strain [1,2], pH [3], humidity [4], plantar pressure [5], accelerometry [6] and physiological solutions [7]. To date, different POF materials have been investigated for sensing applications, such as poly(methyl methacrylate) (PMMA) [8], cyclic olefin copolymers TOPAS [9], cyclic transparent amorphous fluoropolymers CYTOP [10], the cyclic-olefin polymer ZEONEX [11], and polycarbonate (PC) [12,13]. 

PC-based POFs were first proposed in 1986 [14], and since then an increasing number of studies have been carried out on PC-based POFs [12,13,15,16]. PC is an excellent waveguide material, since it is not only transparent to visible light presenting good optical properties, but also yields and breaks at high strains showing good mechanical properties [12]. Moreover, the glass transition temperature (T_g_) of PC is 145 °C, which is one of the highest among optically transparent polymers currently used for POF fabrications, and the maximal water absorption of PC is 0.3% [13]. Compared to standard step-index POFs, microstructured POFs (mPOFs) have drawn great attention since 2001 [17], as the nano-sized fiber microstructures could induce a variety of optical effects, such as single-modeness at all wavelengths [18]. Recently, the solid-core PC mPOFs were fabricated by using a drill-and-draw technique starting from casting of plastic granules [12]. Regarding optical property, the fiber transmission loss was measured to be 8.91 dB/m at 833.5 nm [12] and reduced to 4.06 dB/m at 819 nm by casting improvement [13]. For the mechanical property, the fiber pseudo-yield point was obtained with a strain of 5% with a relatively low Young’s modulus of 3.03 GPa and a high strain of 36.3% at break, which is more than twice as much as PMMA [12].

Based on the advanced properties of PC mPOF, researchers have studied PC mPOF fiber Bragg gratings (FBGs) from inscriptions to applications. First, an FBG was successfully inscribed by He-Cd CW laser at 325 nm using the phase-mask technique. Then, a strain sensitivity of 0.701 pm/με was obtained by linear regression in the range 0–3%, and the temperature sensitivity was measured to be −29.99 pm/°C exhibiting a linear response between 23.6 °C and 125 °C [12]. Additionally, a relative humidity (RH) sensor was characterized with a sensitivity of 7.31 pm/% RH in the range 10–90% RH at 100 °C [13].

Over the last 40 years, many Mach–Zehnder (M–Z) fiber-optic sensors have been proposed and experimentally demonstrated in the sensing of temperature [19,20] and strain [21] using silica optical fibers (SOFs). For example, T. Okamoto et al. developed a fiber-optic M–Z interferometric sensor using multimode fibers. The fringe shift was detected by a spatial filtering detector with a sensitivity of 72.3 fringes/°C per meter (unit fiber length) [19]. S. Gao et al. demonstrated a highly sensitive M–Z interferometric temperature sensor fabricated by a core-offset splicing technique and the refractive index matching liquid was filled in the quartz capillary. As a result, a sensitivity of 21.2 nm/°C was achieved [20]. F. Xu et al. introduced a novel temperature-insensitive strain sensor fabricated by concatenating two waist-enlarged fiber tapers, which were separated by a short piece of photonic crystal fiber. The strain sensitivity was calculated to be 3.02 pm/με [21].

Although SOF-based M–Z interferometric sensors were studied extensively, POF-based ones have been seldom investigated. M. Silva-López et al. set up an M–Z interferometer by splitting a He-Ne laser beam (632.8 nm) using a beam splitter. Then, one beam was coupled into the core of a PMMA step-index fiber, and the other beam propagated in free space. Finally, two beams were recombined by another beam splitter, and the interference fringe variation was detected by a photodetector. By monitoring the variation of the optical phase, the elongation and temperature sensitivities were calculated to be 131 × 10^5^ rad m^−1^ and −212 rad m^−1^ K^−1^, respectively [22]. O. Abdi et al. demonstrated an M–Z interferometer configuration using a single mode PMMA POF with a strain measurement of up to 10%. The phase response of the interferometer was measured with a 3 × 3 coupler interrogator and two photodetectors integrated with a data acquisition system [23]. Although PMMA POF can be used for M–Z interferometric sensors, the glass transition temperature and the elongation at break of the PMMA material are smaller than those for the PC material. These drawbacks of the PMMA material limits the sensing application at higher temperature or larger elongation [13,24].

Here, we report a temperature and a strain sensor combining a PC mPOF and M–Z interferometric technique. The fiber used in this report was manufactured at DTU Fotonik using a drill-and-draw method [12] with a core and cladding diameter of 9 μm and 146 μm, respectively. The average diameter and the pitch of the micro-sized holes are 2.33 μm and 5.83 μm, respectively. Considering the hole-to-pitch ratio to be 0.4, this fiber is an endlessly single mode fiber [25]. This M–Z interferometer is composed of a He-Ne laser, a fiber coupler and two fiber arms. A piece of POF was inserted in one arm with two end-faces butt-connected to SOFs. By monitoring interference fringe shift number, the temperature and strain sensing were characterized with linear response properties. 

Before developing the M–Z interferometric set-up, the POF has to be connected permanently to the SOFs to facilitate later operations. Unlike a silica fiber connection, which can be operated by a commercial fusion splicer, a POF-SOF connection cannot use that machine, because the melting points of the two fiber materials are quite different. To solve this problem, A. Abang et al. fabricated a demountable physical contact ferrule connector between a SOF and a POF. The PMMA-based multimode mPOF has an outer diameter of 150 μm and a core diameter of 50 μm, respectively. First, the end of the POF was etched to a diameter of 120 μm to match the inner diameter of the ceramic ferrule connector; then, the POF was ultraviolet (UV)-cured inside the ferrule, and the ferrule end was polished; finally, the connector was finished and successfully aligned to the multimode SOF with a core diameter of 50 μm showing good performance [26]. It is worth mentioning that the large core size of both fibers provides tolerance to core misalignment in this connectorization method. However, for connectorization between two single-mode fibers with a core diameter of under 10 μm, this method is not applicable, since the POF core is normally not perfectly aligned in the geometric center of the ferrule, and thus the misalignment between cores of the POF and the SOF would result in much higher attenuation and degraded mode coupling [23]. Therefore, before going to the M–Z interferometric sensing part, a connection method by monitoring projecting images is demonstrated. 

## 2. Polymer Optical Fiber-Silica Optical Fiber (POF-SOF) Connectorization

**Figure 1:**
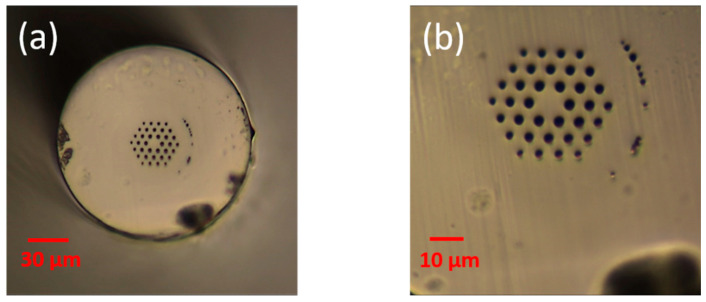
Cross-sectional image of polycarbonate (PC) microstructured polymer optical fiber (mPOF) after cleaving at 80 °C. The endlessly single mode fiber was manufactured at DTU Fotonik with a core diameter of 9 μm and a cladding diameter of 146 with a dimension scale of 30 μm (**a**) and 10 μm (**b**), respectively.

As a result of inexistence of commercial devices for connectorization between POF and SOF pigtails, POF-SOF connectorization has to be operated manually [27,28]. Here, the POF cleaving set-up includes a commercial hot plate (SP-H550, Shenzhen Kejing Star Technology Company, Shenzhen, China) and a sharp blade (Double Edge Shaving Blade, FEATHER Safety Razor Co., Ltd., OSAKA, Japan) with a blade thickness of 0.1 mm. Both the POF and the blade were heated at 80 °C, and then the POF was cleaved by hand slowly. Figure 1 demonstrates the cross-sectional image of the PC mPOF cleaved on the hot plate. The SOF used for connecting with POF is G.652.D single mode fiber at the C + L band, which is commercially available.

**Figure 2:**
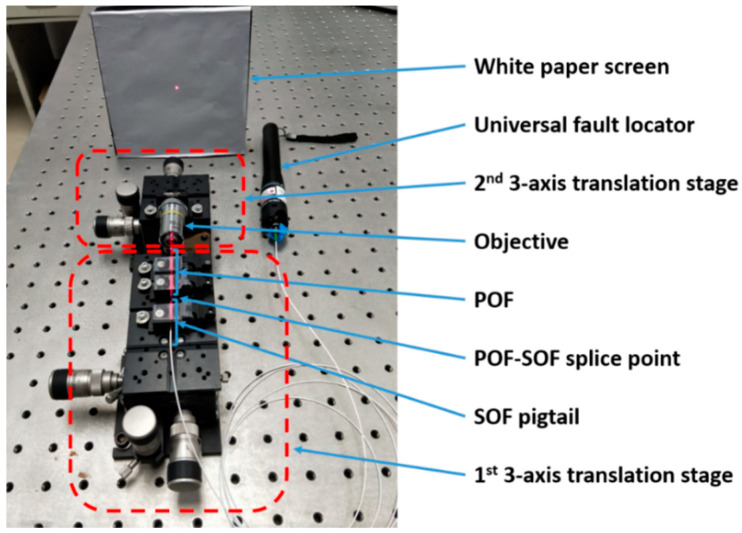
POF-silica optical fiber (SOF) connectorization set-up imaging method including an objective, a red-light universal fault locator, two translation stages and a white paper screen.

The cores of the POFs and SOFs used in this work virtually have similar diameters, which eases the alignment of these fibers and ensures a low connectorization loss. The UV glue, acting as a refractive index matching agent, alleviates the Fabry–Perot effect. The POF and the SOF were connectorized by a three-axis translation stage (MBT616D/M, Thorlabs). By the imaging projecting method with the help of an objective (10X, Edmund Optics) mounted on another three-axis translation stage, a red-light universal fault locator and a white paper screen, two fibers were well aligned. Figure 2 demonstrates the POF-SOP connectorization set-up. Thanks to this, the single mode mPOF with a small core can be easily aligned with a single mode SOF. Before fiber alignment, a tiny amount of UV glue (Norland 86H) was smeared on the SOF end-face. 

**Figure 3:**
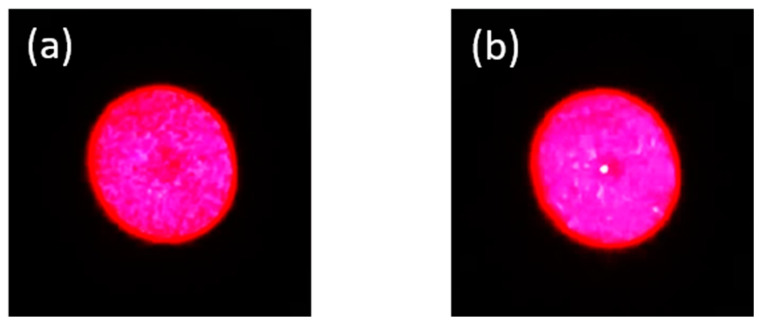
The PC mPOF end-face images through an objective illuminated by the red light. (**a**) Misalignment image with a uniform end-face. (**b**) The POF and the SOF are well aligned with a brighter point in the center of the fiber end-face image.

The image on the screen was captured by a camera in a dark environment. The dimension of the image varies with the distance between the fiber output end and the objective lens. Figure 3a illustrates a uniform end-face image of the PC mPOF on the screen, because a mismatch between two fibers existed and consequently light was not well coupled in the core; however, in Figure 3b, a brighter point was presented in the center of the image, so we believe that more power was coupled in the core and fibers were well aligned. When coupling the other end of the POF to another SOF for transmission measurement, transmitted power is regarded as a reference, as no image could be observed in this case. When the transmitted power reaches the maximum, both fibers are well aligned. Finally, the splicing region was irradiated by a UV light at 365 nm. The UV curing process was conducted step by step to ensure high stability and strength of the splice. It is worth mentioning that although only mPOFs were used in this work to perform POF-SOF connectorization, this technique can be extended to step-index POFs as well. Also, several samples were prepared using this method for temperature and strain measurement. 

## 3. Sensing Principle and Experimental Set-Up 

The sensing mechanism of the M–Z interferometer can be described simply as follows. When the effective refractive index n or the length l of the POF is changed, phase difference δφ of the light propagating in the fiber core can be calculated as $\delta \varphi =k_{0}\left(l\cdot \delta n+\delta l\cdot n\right)$. $k_{0}$ is wavenumber defined as $2\pi /\lambda_{0}\;\;$, where $\lambda_{0}$ is the operating wavelength in vacuum. When a coherent laser beam in one arm propagating in the POF core recombines with the other coherent laser beam in the other arm, transmitted light from both arms would partially overlap and produce interference fringes that would shift in response to variations in temperature or strain. The fringe shift number is expressed as:

(1) $$N=\delta \varphi /\left(2\pi \right)=\left(l\cdot \delta n+\delta l\cdot n\right)/\lambda_{0}.$$

Furthermore, fringe shift versus temperature can be expressed as:

(2) $$\frac{dN}{dT}=\left(l\frac{dn}{dT}+n\frac{dl}{dT}\right)\frac{1}{\lambda_{0}}=ln\left(\frac{dn}{dT}\frac{1}{n}+\frac{dl}{dT}\frac{1}{l}\right)\frac{1}{\lambda_{0}}=ln\left(\alpha_{n}\frac{1}{n}+\alpha_{l}\right)\frac{1}{\lambda_{0}}.$$

$\alpha_{n}$ is the thermo-optic coefficient (TOC), and $\alpha_{l}$ is the coefficient of thermal expansion (CTE). Moreover, fringe shift versus strain ε can be expressed as:

(3) $$\frac{dN}{d\varepsilon }=l\left(\frac{dn}{d\varepsilon }+n\frac{dl}{d\varepsilon }\right)\frac{1}{\lambda_{0}}=ln\left(\frac{dn}{d\varepsilon }\frac{1}{n}+\frac{dl}{d\varepsilon }\frac{1}{l}\right)\frac{1}{\lambda_{0}}=ln\left(-p_{e}+1\right).$$

$p_{e}$ is the strain-optic constant of the material. 

**Figure 4:**
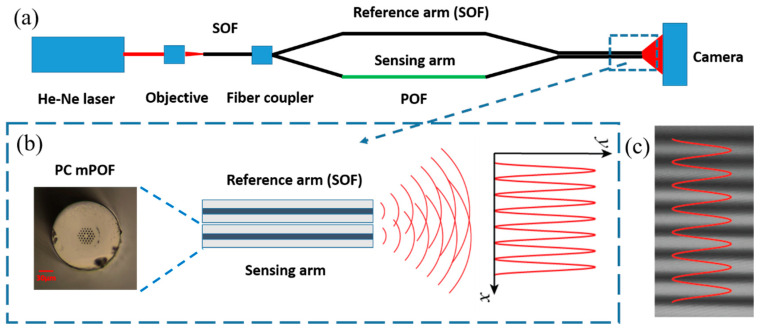
(**a**) Mach–Zehnder (M–Z) interferometer configuration for sensing application. The wavelength of the He-Ne laser is 632.8 nm. (**b**) Close-up of the parallel fiber positioning and the demonstration of the interference fringe generation. (**c**) Real interference fringe pattern recorded by the camera.

To study temperature and strain characterization based on POFs and the interferometric technique, we set up an M–Z interferometer, as shown in Figure 4a. Light from a He-Ne laser was first coupled into a SOF by a 10 X objective; then the red light propagating along the fiber was divided into two arms (a reference arm and a sensing arm) by a 1 × 2 fiber coupler; and the reference arm is composed of a piece of SOF, while the sensing arm is composed of two pieces of SOF with a piece of POF in between. Finally, the SOFs of the two arms at the output end were immobilized in parallel for interference fringe generation, as illustrated in Figure 4b. The experimental interference fringe pattern was recorded by the camera. This diagram is similar to that for the gas refractometer [29]. It is worth mentioning that for mPOF with a short length, even the condition for endlessly single-mode guidance is fulfilled, higher order modes could be observed [30]. In addition, the SOF used in this work is multimoded at 632.8 nm, although the fiber is single-moded at C+L band. Thus, the interference fringe pattern is modulated by a speckle pattern [19]; however, for a single speckle, the fringe pattern is uniform, as shown in Figure 4c. This speckle effect could be removed by using a laser source with a longer wavelength where both the mPOF and the SOF are single mode.

**Figure 5:**
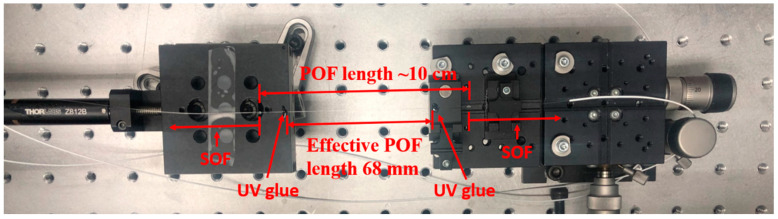
M–Z interferometer configuration for strain application. The effective POF length is 68 mm.

Both temperature and strain-sensing experiments were conducted inside a room with temperature and relative humidity (RH) under control. The temperature and RH were kept constant at 26 °C and 40%, respectively. For the temperature sensing application, a piece of PC mPOF was positioned on the surface of a temperature-controlled breadboard (PTC1/M, Thorlabs) with a temperature stability of 0.1 °C, and a sponge was then used to cover the breadboard and the fiber as a thermal insulation material to reduce thermal perturbation from the ambient environment. For strain-sensing applications, the strain-controlled set-up for the POF in the sensing arm was depicted in Figure 5, which is part of the M–Z interferometer configuration in Figure 4a. In this sensing arm, a piece of POF (~10 cm) was cascaded between two SOFs. To avoid the stretch on the connection joint between the POF and the SOF, the effective sensing length of the POF was shortened to 68 mm, which was measured between two UV-glued points for the fiber fixation as shown in Figure 5. The left end of the POF could be pulled by a one-axis translation stage (MT1/M, Thorlabs), whose position was controlled by a motorized actuator (Z812B, Thorlabs) with a minimum resolution of ~29 nm. The right end of POF was immobilized by a fiber clamp. The whole strain sensing set-up was placed in an optical enclosure to reduce the impact of the external environment.

## 4. Temperature Measurement Result and Discussion

**Figure 6:**
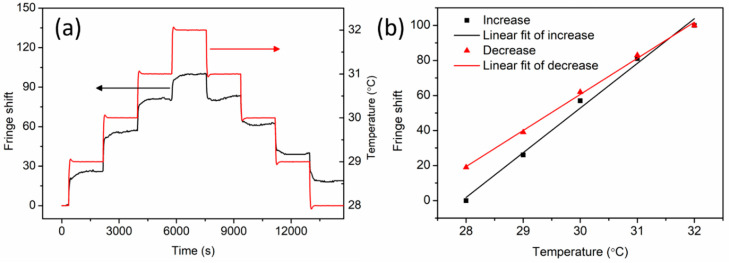
(**a**) Fringe and temperature variation as a function of time and (**b**) linear fit of fringe shift versus temperature (increase and decrease) for non-annealed PC mPOF.

First, an 18.8-cm-long piece of non-annealed PC mPOF was positioned on the surface of the breadboard without pre-strain control, and the temperature measurement was performed by counting fringe shifts, shown in Figure 4c, in the temperature range from 28 °C to 32 °C. After stabilization at 28 °C, the temperature was adjusted by steps of 1 °C (first increase, then decrease). The temperature response time to reach the set value is ~4 min. Meanwhile, the fringe shifts varied accordingly as a function of temperature, as shown in Figure 6a. After 30 min of each temperature adjustment, fringe shifts were adopted for temperature sensitivity linear regressions, as illustrated in Figure 6b. The computed temperature sensitivity was 25.5 fringes/°C ± 1.2 fringes/°C for the increasing process. The converted sensitivity per unit length is 135.6 fringes/°C/m, which is twice as much as the silica fiber [19]. It is worth mentioning that although the TOC for PC (–14.3 × 10^−5^/°C) [24] is around 18 times as high as that for silica (7.97 × 10^−6^/°C) [31], the sensitivity difference is not that case accordingly. It is because of the fact that, for silica fiber, both TOC and CTE are positive, which are 7.97 × 10^−6^/°C and 5.5 × 10^−7^/°C, respectively [31]; however, for PC mPOF, TOC and CTE are the contrary, with a negative TOC of –14.3 × 10^−5^/°C and a positive CTE of 7 × 10^−5^/°C [24]. The optical phase change per meter versus fiber temperature is 852.2 rad/°C/m, which is more than four times as much as 212 rad/°C/m for the PMMA fiber [22]. This significant difference may mainly be attributed to the TOC difference between PC (−14.3 × 10^−5^/°C) and PMMA (−8.5 × 10^−5^/°C) materials, as the CTEs of the two materials are quite similar (7 × 10^−5^/°C for PC and 6.5 × 10^−5^/°C for PMMA) [24]. In addition to temperature increasing measurement, the sensitivity decreased to 20.6 fringes/°C ± 0.6 fringes/°C for decreasing process. The higher sensitivity for temperature increasing process could be attributed to the permanent shrinkage effect of the PC mPOF arising from polymer chain relaxation at higher temperatures [32]. According to Equation (2), both negative TOC and positive CTE of PC material contribute to optical phase change, but the shrinkage reduces the fiber thermal expansion. However, when temperature decreases, the shrinkage effect resulted from polymer chain relaxation disappears, and both negative TOC and positive CTE influence optical phase change in accordance with Equation (2). 

**Figure 7:**
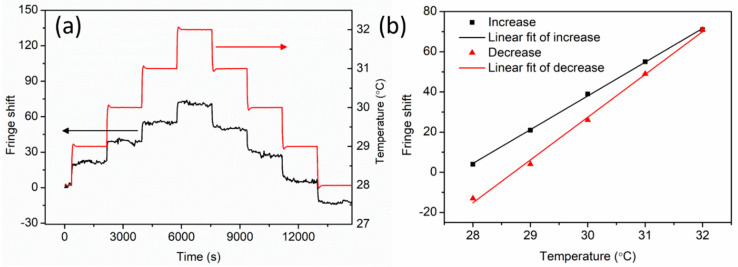
(**a**) Fringe and temperature variation as a function of time and (**b**) linear fit of fringe shift versus temperature (increase and decrease) right after PC mPOF annealing at 125 °C for 36 h.

Then, the fiber was annealed at 125 °C for 36 h to reduce the frozen-in stress arising from the fiber drawing process [13]. After that, the temperature experiment was repeated immediately on the same day with the same procedure, and the results were shown in Figure 7. It is found that the fringe shift fluctuates during the whole measurement. By linear regression, the sensitivities were 16.8 fringes/°C ± 0.2 fringes/°C and 21.3 fringes/°C ± 0.6 fringes/°C for increasing and decreasing process, respectively. In this case, the temperature sensitivity for the increasing process is lower than that for the decreasing process, which is opposite to the non-annealed fiber temperature measurement. This phenomenon may be attributed to polymer viscoelastic properties [33] as well as residual stress in the fiber, and thus POFs need more time for stress relaxation during the decreasing temperature process. This hysteresis effect could be reduced by applying a pre-strained fiber [32] or a fixed fiber on a plate [33], which could reduce the temperature response time by attenuating the thermal expansion contribution. In addition to temperature response linearity improvement, the sensitivity can also be improved due to the non-existence of the CTE term in Equation (2). 

**Figure 8:**
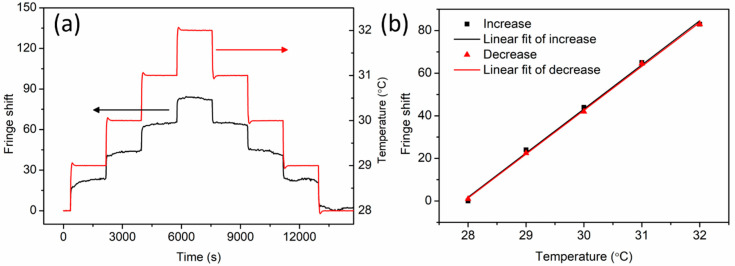
(**a**) Fringe and temperature variation as a function of time and (**b**) linear fit of fringe shift versus temperature (increase and decrease) one month after PC mPOF annealing at 125 °C for 36 h.

One month later, the same experiment with the annealed fiber was carried out again. As illustrated in Figure 8, the fluctuation was reduced dramatically with temperature sensitivities of 20.7 fringes/°C ± 0.6 fringes/°C and 20.6 fringes/°C ± 0.3 fringes/°C for increasing and decreasing process, respectively. The reduction of fluctuation and the improvement of linear response could be attributed to adequate residual stress relaxation during the period after annealing. The temperature measurement in the small range from 28 °C to 32 °C is due to the ease of image data acquisition and processing. The upper limit of the measuring range can be as high as 125 °C at which the polymer fiber is annealed. Measurements for higher temperatures will be implemented in the near future.

## 5. Strain Measurement Results and Discussion

**Figure 9:**
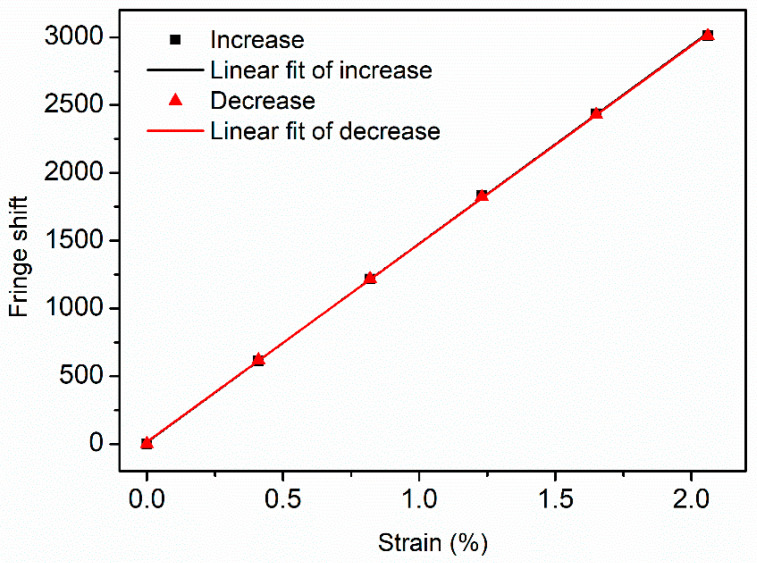
Fringe shift versus axial strain (increase and decrease) for non-annealed PC mPOF with linear response.

Axial strain characterization of non-annealed PC mPOF was then performed by recording fringe shifts with strain change up to 2.06%. The strain values were expressed and calculated by the ratio between the POF length change and the initial operating length (68 mm). The displacement speed was 0.003 mm/s controlled by the motorized actuator with steps of 0.28 mm. A very low speed was adopted for the ease of fringe shift counting from the recorded video. After each step of movement, the fringe was stabilized for 20 s to ensure a stable fringe. Figure 9 displays the results for forward and reverse strain. The forward and reverse sensitivity are 1465 fringes/%ε ± 9 fringes/%ε and 1461 fringes/%ε ± 7 fringes/%ε, respectively, calculated by linear regression, exhibiting a linear response over the strain range 0–2.06%. Since the fiber pseudo-yield point is 5% in strain according to Ref. [12], the strain sensing range can be extended accordingly.

For comparison, the sensitivity is converted to 135 × 10^5^ rad/m (optical phase change versus fiber displacement), which is a little bit higher than 131 × 10^5^ rad/m for the PMMA fiber [22]. Since the Young’s modulus of the PC mPOF was estimated to be 3.03 GPa [12], the fringe shift versus fiber axial force sensitivity was calculated to be ~2886 fringe/N according to Equation ε=σ/E=F/(E·s), where ε, σ, *E*, *F*, *s* are the strain, stress, Young’s modulus, force and transverse area, respectively. The force measurement resolution is calculated to be 3.47 × 10^−4^ N corresponding to one fringe shift. Supposing that a primitive electronic circuit is used to resolve a change between the maximum and the minimum of the fringe intensity, the force measurement resolution could be doubled. Furthermore, by using a slit and a single photodiode, around two orders of resolution improvement could be gained by analyzing full intensity curves, similar to the gas refractometer methodology reported by H. Chen et al. [34]. Finally, the force resolution could reach the order of 10^−6^ N, which is higher the that based on FBG technology using non-etched PMMA POFs [1]. In that work, the axial force sensitivity is 25.55 nm/N with a converted force resolution of 3.91 × 10^−5^ N, assuming that the resolution of the optical spectrum analyzer (OSA) is 1 pm. 

## 6. Conclusions

We presented a convenient method for fiber connectorization between POFs and SOFs. Then, based on this technique, the PC mPOF was inserted in one sensing arm of the fiber Mach–Zehnder interferometer for temperature and strain-sensing application. By monitoring the interference fringe shift, the temperature sensitivity based on non-annealed fiber was found to be 25.5 fringes/°C for increasing temperature and 20.6 fringes/°C for decreasing temperature over the temperature range 28–32 °C. Just after fiber annealing, the temperature sensitivities were measured to be 16.8 fringes/°C and 21.3 fringes/°C for increasing and decreasing process, respectively. One month later, the linear response was improved with a temperature sensitivity of ~20.7 fringes/°C in the forward and the reverse process. For strain measurement based on non-annealed fiber, the strain was found to be ~1463 fringes/%ε exhibiting a linear response over the strain range of 0–2.06%. In addition, the fiber axial force sensitivity was calculated to be ~2886 fringes/N, converted to a force detection resolution of ~3.47 × 10^−4^ N. This work shows a couple of advantages, such as very low cost, high sensitivity, straightforward sensing mechanism and ease of fabrication.
